# Genetic Stability, Phenolic, Flavonoid, Ferulic Acid Contents, and Antioxidant Activity of Micropropagated *Lycium schweinfurthii* Plants

**DOI:** 10.3390/plants10102089

**Published:** 2021-10-01

**Authors:** Diaa Mamdouh, Hany A. M. Mahgoub, Ahmed M. M. Gabr, Emad A. Ewais, Iryna Smetanska

**Affiliations:** 1 Department of Plant Food Processing, Agricultural Faculty, University of Applied Sciences Weihensteph-an-Triesdorf, Markgrafenstr 16, 91746 Weidenbach, Germany; 2 Botany & Microbiology Department, Faculty of Science, Al-Azhar University, Nasr City, Cairo 11884, Egypt; h.mahgoub@azhar.edu.eg (H.A.M.M.); ewais_e@yahoo.com (E.A.E.); 3 Department of Plant Biotechnology, Genetic Engineering and Biotechnology Research Division, National Research Centre (NRC), Cairo 12622, Egypt; ahmedgabr01@gmail.com

**Keywords:** *Lycium schweinfurthii*, micropropagation, genetic stability, ISSR-PCR, RAPD-PCR, SDS-PAGE, HPTLC, DPPH, ABTS

## Abstract

*Lycium schweinfurthii* is a Mediterranean wild shrub rich in plant secondary metabolites. In vitro propagation of this plant may support the production of valuable dietary supplements for humanity, introduction of it to the world market, and opportunities for further studies. The presented study aimed to introduce an efficient and reproducible protocol for in vitro micropropagation of *L. schweinfurthii* and assess the genetic stability of micropropagated plants (MiPs) as well as to estimate phenolic, flavonoid, ferulic acid contents, and the antioxidant activity in leaves of micropropagated plants. Two DNA-based techniques, random amplified polymorphic DNA (RAPD) and inter-simple sequence repeats (ISSR), and one biochemical technique, sodium dodecyl sulfate-polyacrylamide gel electrophoresis (SDS-PAGE), were used to assess the genetic stability in MiPs. Spectrophotometric analysis was performed to estimate total phenolic and flavonoid contents and antioxidant activity of MiPs leaves, while ferulic acid content was estimated using high-performance thin-layer chromatography (HPTLC). Sufficient shoot proliferation was achieved at MS (Murashige and Skoog) medium supplemented with 0.4 mg L^−1^ kinetin and rooted successfully on half-strength MS medium fortified with 0.4 mg L^−1^ Indole-3-butyric acid (IBA). The Jaccard’s similarity coefficients detected in MiPs reached 52%, 55%, and 82% in the RAPD, ISSR, and SDS-PAGE analyses, respectively. In the dried leaves of MiPs, the phenolic, flavonoid, and ferulic acid contents of 11.53 mg gallic acid equivalent, 12.99 mg catechin equivalent, and 45.52 mg were estimated per gram, respectively. However, an IC_50_ of 0.43, and 1.99 mg mL^−1^ of MiP dried leaves’ methanolic extract was required to scavenge half of the DPPH, and ABTS free radicals, respectively. The study presented a successful protocol for in vitro propagation of a valued promising plant source of phenolic compounds.

## 1. Introduction

One member of the Solanaceae (the nightshade) family is the genus Lycium, comprising more than 70 species and which has a disjunctive distribution in temperate to subtropical regions in South America, North America, Africa, Eurasia, and Australia [1]. Within buckthorns (Lycium), *Lycium schweinfurthii* is grouped according to phylogenetic studies in a clade with other Old World species of the genus. Within this clade, this species is closely related to *L. acutifolium*, *L. eenii*, *L. shawii*, *L. bosciifolium*, *L. hirsutum*, and *L. villosum*. The species is sometimes put to *L. intricatum* [2]. *L. schweinfurthii* grows in temperate climates and is well spread throughout the southern Mediterranean region as well as in Egypt, Algeria, Tunisia, and Libya [3]. *L. schweinfurthii* is distributed in Egypt in the great south-western desert, northern coastal region [4], and islands of Lake Burullus [3]. The plant is a 2–3 m high, rigid, upright shrub with a spiny stem. Its leaves are succulent and hairless that are 12–20 mm long and 2–4 mm wide and arranged in alternate patterns (one leaf per node) while its flowers are hermaphrodite. The fruit is a black, spherical, sometimes egg-shaped berry that measures 4–5 mm in diameter [5]. *L. schweinfurthii* suffers from different types of threats that affect its distribution, whether natural or caused by human activities, i.e., soil fragmentation, cutting, grazing, and firing [3].

It is difficult in many seasons to obtain seeds or crops from wild plants, especially with their small number and wide geographical distribution, as in *L. schweinfurthii*. Hence, it is imperative to micropropagate plants in vitro to maintain the explant source at all times of the year. For decades, the micropropagation of plants was the only technique that maintained and promoted the economic value of many agricultural species [6]. Furthermore, it is an efficient technique for in vitro multiplication of endangered species, e.g., *Magnolia sirindhorniae* [7], as well as for producing secondary metabolites, e.g., *Eryngium alpinum* L. [8]. Although no reports were found on the micropropagation of *L. schweinfurthii*, it is well studied in other species of the Lycium genus. Multiple shoots and adventitious buds of *L. ruthenicum* were developed in vitro not only from stems but also from leaf explants [9]. Moreover, the best shoot proliferation of *L. depressum* was achieved at a low concentration of BA (6-benzyl adenine) and rooted in full-strength MS medium (Murashige and Skoog medium) supplemented with IBA (indole-3-butyric acid) with a high survival rate [10]. Micropropagation protocols were also developed in *L. barbarum* [11] and *L. chinense* [12]. 

To maintain the effectiveness of in vitro propagation, genetic stability must be ensured, especially with successive generations. Diverse techniques are used to determine the genetic stability of regenerated plants in terms of plant genomes or transcribed proteins. One of these is the random amplified polymorphic DNA (RAPD) PCR technique, which is a rapid, inexpensive, and simple method for detecting genetic differences as it does not require any previous information about the plant genome [13]. RAPD-PCR was used to determine the genetic stability in micropropagated plants of *Prunus salicina* [14], *Echinacea purpurea* [15], *Dendrobium fimbriatum* [16], and *Rhynchostylis retusa* [17]. A more specific technique than RAPD is the inter-simple sequence repeats (ISSR) PCR technique. It is an efficient, quick, and reproducible technique in which the targets are the DNA fragments located between adjacent microsatellite regions, while the RAPD-PCR targets are random [18]. Wójcik et al. [19] used ISSR primers to observe the genetic stability of regenerated plants of *Ribes grossularia* L. Both techniques are used together to obtain more realistic and accurate results [20,21,22]. 

Otherwise, the differences in the protein profile of the regenerated plants also reflect the extent of genetic stability at the level of gene expression. Sodium dodecyl sulfate-polyacrylamide gel electrophoresis (SDS-PAGE) is a technique used to show the differences in the transcribed polypeptides in the micropropagated plants concerning the mother plant [23,24]. The SDS-PAGE technique was assessed to check the genetic stability of in vitro micropropagated plants of *Pilosocereus robinii* [24], *Musa* spp. [25], and *Phoenix dactylifera* L. [26]. At both levels, the DNA genome and the transcribed proteins are essential for recognizing the genetic stability in the regenerated plants of *L. schweinfurthii*. 

The functional effect of certain plant species and their use in folk medicine depend mainly on their active secondary metabolites [27,28]. Plants of *L. schweinfurthii* have been reported to contain a high level of phenolic compounds, particularly flavonoids [29]. These secondary metabolites play a major role in adapting the plant to the environment and maintaining its survival [30]. Flavonoids are naturally produced phenolic compounds in plants and play an important role in the protection against unfavorable environmental conditions such as drought [31], high concentrations of aluminum in soil [32], UV-irradiation [33], and defense plants against herbivores, bacteria, and fungi [34]. Phenolic compounds have a role in modern human therapy, e.g., controlling hyperglycemia associated with type 2 diabetes at early stages when included in the human diet [35]. Moreover, flavonoids are reported to protect humans against numerous diseases due to the fact of their strong anti-oxidative [36], anti-inflammatory [37], anticarcinogenic [38], antiviral [39], and antibacterial [40] activities as well as a direct cytoprotective effect on several human systems (i.e., coronary and vascular systems) and organs (i.e., liver and pancreas) [41,42]. These features put them among the most attractive natural substances available for enhancing the options of the previously mentioned therapy [43]. The leaves of *L. schweinfurthii* contain large quantities of flavonoids compared to roots, stems, and flowers [44]. The main phenolics found in leaves are quercetin, kaempferol, gallic acid, ferulic acid, and apigenin [29]. Six glucosides have been isolated from *L. schweinfurthii*. Four of them showed a potent inhibitory activity that could decrease postprandial hyperglycemia in diabetic patients [45].

Although many plants contain high-value phenolic compounds, it is difficult to cultivate at a large-scale due to the specific ecological conditions. Corresponding plant in vitro cultures, particularly plant cell cultures, provide an attractive alternative source of phenolics that can overcome the limitations of extracting useful metabolites from limited natural resources [46]. Obtaining phenolic compounds from plant’s in vitro cultures is one of the more interesting research areas in recent decades due to the fact of their benefits. Phenolic content can be elevated in culture medium such as in *Zingiber officinale* Rosc. [47], *Sequoia sempervirens* [48], *Rosa damascene* Mill [49], and grape [50].

It is worth searching for alternative plant sources to meet the nutritional needs of humans and to protect them from diseases resulting from malnutrition and a lack of functional substances in the future. Thus, the present study is the first attempt to optimize a protocol for direct in vitro plant regeneration in *L. schweinfurthii* as well as to evaluate its phenolic, flavonoid, ferulic acid contents, and antioxidant activity of in vitro leaves’ extract.

## 2. Materials and Methods

### 2.1. Plant Material and Culture Conditions

Fruits of *L. schweinfurthii* were collected during March 2016 from Jazirat Al-Kawm Al-Akhdar (the green islet) located in Burullus Lake (northern Nile Delta), Egypt. The fruits were air-dried for approximately 120 h, and then their envelopes were removed to obtain their seeds. The plant seeds were washed with 70% ethanol for 30 s, and then they were surface sterilized by soaking in 30% commercial Clorox for 10 min. Seeds were washed with sterilized distilled water 4 times to remove the remaining bleach.

After the sterilization process, seeds were cultured in 300 mL jars containing 30 mL basal MS medium, including vitamins (Caisson Labs, Smithfield, UT, USA), with 3% sucrose and solidified using 7% agar (ROTH Company, Carlsruhe, Germany) and incubated at 23 ± 2 °C under a 16 h photoperiod of 2500 lux by cool fluorescent lamps.

### 2.2. In Vitro Micropropagation

For vegetative propagation, nodal segments were cut and cultivated on full-strength MS media including vitamins supplemented with BA (0.4, 0.8, 1.6, or 3.2 mg L^−1^), kinetin (0.4, 0.8, 1.6, or 3.2 mg L^−1^), BA + Kin (0.2 + 0.2, 0.4 + 0.4, 0.8 + 0.8, or 1.6 + 1.6 mg L^−1^), or BA + Kin + NAA (0.2 + 0.2 + 0.2 or 0.4 + 0.4 + 0.4 mg L^−1^) and on basal MS medium as a control. Seven nodal explants were used for shoot formation in each treatment. Regenerated shootlets were then transferred to basal full-strength MS, half-strength MS, half-strength MS medium fortified with NAA (0.4, 0.8, or 1.6 mg L^−1^) or IBA (0.4, 0.8, or 1.6 mg L^−1^). To determine the rooting capacity and the most suitable rooting medium, eight shootlets were used in each treatment.

### 2.3. DNA Extraction and PCR Amplification Conditions

Total DNA was extracted from leaves of two in vitro mother plants and their micropropagated plantlets for three generations using the E.Z.N.A. kit (VWR, Darmstadt, Germany). Twelve primers (i.e., 7 RAPD and 5 ISSR) out of a total of twenty primers (Thermo Fisher, Frankfurt, Germany) were selected to amplify DNA fragments. The protocol for RAPD and ISSR analysis was adapted from Martins et al. [51] and Williams et al. [52]. PCR was performed in a volume of 20 µL using Invitrogen™ Platinum™ master mix (Thermo Fisher, Frankfurt, Germany). The amplification reaction consisted of an initial denaturation step at 94 °C for 5 min, followed by 43 cycles of 1 min at 92 °C, 1 min at a specific annealing temperature (Table 1), and 2 min at 72 °C; there was one last extension step of 7 min at 72 °C. Amplifications were performed in a Bio-Rad T100™ thermal cycler (Bio-Rad Laboratories, Hercules, CA, USA) for both RAPD and ISSR. DNA amplification fragments were separated with 1.5% agarose gel using 1x TBE buffer and stained with Red-Safe™ nucleic acid staining solution. Gels were then analyzed with CAMAG^®^ TLC Visualizer 2 (CAMAG, Muttenz, Switzerland).

### 2.4. Protein Extraction and SDS-PAGE

Total protein was extracted from the healthy leaves of two in vitro mother plants and their micropropagated plantlets for three generations. Ten milligrams of ground, fine powder were homogenized thoroughly with a 400 μL extraction buffer (0.6 g Tris base, 0.2 g sodium dodecyl sulfate (SDS), 30 g of urea, and 1 mL β-mercaptoethanol in 100 mL double-distilled water) using vortex. The mixture was centrifuged at 13,000 rpm for 10 min at room temperature after keeping overnight at 4 °C. Twenty microliters of the extracted protein samples were boiled in a water bath for 3–5 min before loading them on the gel. SDS-PAGE was performed according to Laemmli [53] using 12.5% resolving gel, 4% stacking gel, and bromophenol blue as a tracking dye. After carrying out the electrophoresis at 150 volts and 25 milliamperes, the gel was de-stained in a methanol:glacial acetic acid:water (4:1:5) mixture. Then, it was kept overnight in Coomassie Brilliant Blue buffer for staining. The gel was photographed, and the molecular weights of the polypeptide bands were estimated against protein molecular weight marker.

### 2.5. Secondary Metabolites

#### 2.5.1. Sample Preparation and Extraction

Leaves of micropropagated plants were randomly collected, freeze-dried, and ground. One gram was collected and Soxhlet extracted with 200 mL of 80% aqueous methanol for 24 h. The extract was concentrated with a rotary evaporator to a concentration of 50 mg mL^−1^ which was then subjected to estimate the phenolic and flavonoid contents as well as the antioxidant activity. More diluted leaves’ extract of 10 mg mL^−1^ was used to quantify the ferulic acid content through HPTLC (high-performance thin layer chromatography) analysis.

#### 2.5.2. Total Phenolic Assay

The total phenolic content of the leaves was determined using the Folin–Ciocalteu assay as described by Marinova et al. [54] with some modifications. An aliquot (200 µL) of extracts or gallic acid (Sigma–Aldrich, St. Louis, MO, USA) standard solution (10, 20, 30, 40, 50, and 100 mg L^−1^) was added to a 5 mL Eppendorf tube containing 1.8 mL distilled deionized water. Two hundred microliters of Folin-Ciocalteu’s reagent (Merck, Schnelldorf, Germany) were added to the mixture and shaken. After 5 min, 2 mL of 7% sodium carbonate (VWR chemicals, Darmstadt, Germany) solution was added and mixed thoroughly. The mixture was diluted to 5 mL with distilled water and incubated for 90 min in the dark at room temperature. The absorbance against the reagent blank was determined at 750 nm with an Analytic Jena Specord^®^ 250 Plus UV-Vis spectrophotometer. Total phenolic content is expressed as mg GAE g^−1^ DW (mg gallic acid equivalents/g dry weight) and calculated as follows: T = CV/M, where T is the total phenolic content, C is the concentration of gallic acid estimated in mg mL^−1^, V is the volume of extract solution in mL, and M is the weight of extract in g.

#### 2.5.3. Total Flavonoid Assay

Total flavonoid content was measured using the aluminum chloride assay as described by Marinova et al. [54] with some modifications. An aliquot (500 µL) of extracts or catechin standard (Sigma–Aldrich, St. Louis, MO, USA) solution (10, 20, 30, 40, 50, and 100 mg L^−1^) was added to a 5 mL Eppendorf tube, containing 2 mL distilled water. To the diluted sample, 150 µL of 5% sodium nitrite (AppliChem, Darmstadt, Germany) was added. After 5 min, 150 µL of 10% aluminum chloride (Carl-Roth, Carlsruhe, Germany) was added. At the sixth min, 1 mL of 1 M sodium hydroxide was added, and the total volume was diluted to 5 mL using distilled water. The absorbance was measured against reagent blank at 510 nm, and total flavonoids were expressed as mg CE g^−1^ DW (mg catechin equivalent/g dry weight) and calculated by the equation: T = CV/M, where T is the total flavonoid content, C is the concentration of catechin estimated in mg mL^−1^, V is the volume of extract solution in ml, and M is the weight of extract in g.

#### 2.5.4. HPTLC Conditions

The high-performance thin-layer chromatography (HPTLC) system (Camag, Muttenz, Switzerland) consisted of a Limomat 5 connected to compressed air, an Automatic Developing Chamber 2 (ADC 2), and a TLC Visualizer 2 supported with visionCATS software. An analytical grade of ferulic acid (Merck, Germany) was used to prepare 400 µg ml^−1^ in methanol as a calibration standard against dry leaves’ extracts of micropropagated plants. TLC silica gel 60 F_254_ aluminum plates (10 × 20 cm, Merck, Darmstadt, Germany) were used for the TLC analysis. Standard and samples were applied to plates as 8 mm bands, 8 mm from the bottom edge of the layer, using Linomat 5. A ferulic acid standard solution of 400 µg ml^−1^ of a volume of 2–9 µL was applied against 2, 4, 6, 8, 10, 12, and 14 µL of dry leaves’ extract. A mixture of ethyl acetate/methanol/water (100:13.5:10, *v*/*v*/*v*) was used as the mobile phase. Plates were developed at room temperature and 60% humidity in an ADC2 automated development chamber. The migration distance of the mobile phase was 70 mm with a development time of 9 min. After development, the chromatogram was visualized and photographed by Visualizer 2 at 254 and 366 nm. The ferulic acid content in the samples was expressed as mg g^−1^ DW.

### 2.6. Antioxidant Capacity

The antioxidant capacity of the micropropagated leaves’ extract was measured using the DPPH (diphenyl-1-picryl-hydrazyl) assay according to Olalere et al. [55] and the ABTS (2,2′-azino-bis(3-ethylbenzothiazoline-6-sulphonic acid)) assay according to Gabr et al. [56].

#### 2.6.1. DPPH Free Radical Scavenging

DPPH is highly sensitive in detecting small differences in antioxidant activities. It is a stable free radical that can accept a hydrogen radical or an electron to convert to a stable molecule. The stock solution of DPPH reagent (1 mM) was prepared and stored at –20 °C until use. The working solution (0.06 mM) was prepared to obtain an absorbance value of 0.8 ± 0.04 at 515 nm. Ten different extract concentrations of micropropagated leaves (between 0.25 and 0.7 mg mL^−1^) were prepared. The absorbance at 515 nm (A_1_) was measured for a mixture of 0.5 mL of each extract concentration with 2.5 mL DPPH working solution after incubation in the dark at room temperature for 30 min. Ethanol was used instead of extract to obtain the absorbance of the control reaction (A_0_). The DPPH radical scavenging activity percentage was calculated as follows: $\left(\left(A_{0}-A_{1}\right)/A_{0}\right)\times 100$. The inhibition percentage was plotted against the different concentrations of the leaves’ extracts to generate a straight-line equation. The extract concentration required to scavenge half of the DPPH radicals (IC_50_) was then determined.

#### 2.6.2. ABTS Free Radical Scavenging

A 7 mM ABTS solution was reacted with 2.4 mM potassium persulphate solution at a ratio of 1:1 (*v*/*v*). The solution was incubated in the dark at room temperature for 16 h. One milliliter of the prepared ABTS^+^ solution was diluted with 60 mL methanol resulting in a working solution with an absorbance of 0.60 ± 0.01 at 728 nm. Fourteen different extract concentrations of micropropagated leaves (between 1.0 and 5.5 mg mL^−1^) were prepared. The absorbance at 734 nm (A_1_) was measured for a mixture of 40 µL of each extract concentration with 1.96 mL blue-green ABTS^+^ working solution after incubation in the dark at 37 °C for 10 min. The control reaction (A_0_), which contains all reagents except the test compound, was run identically. The ABTS^+^ radical scavenging activity percentage was calculated as follows: $\left(\left(A_{0}-A_{1}\right)/A_{0}\right)\times 100$. The inhibition percentage was plotted against the different concentrations of the dry leaves’ extracts to generate a straight-line equation. The concentration of extract required to scavenge half of the ABTS^+^ radicals (IC_50_) was then determined.

### 2.7. Recording Data and Statistical Analysis

The number of plantlets, leaves, distinct nodes, and shootlet length were estimated and recorded after five weeks of cultivation. Recorded data were subjected to statistical analysis of variance (ANOVA) using SigmaPlot v.12.5. The Shapiro–Wilk normality test failed for all data and also for the transformations in the number of plantlets, nodes, roots, and root length. Then, the power of the performed test decreased from 0.50 to 0.001. The normality test passed in shootlet length and passed in the number of leaves after transformation into the square root. The Holm–Sidak method was applied for pairwise comparisons.

RAPD, ISSR, and SDS-PAGE data were scored for presence (1) and absence (0). Three matrices were generated, one for each analysis type. The genetic similarities were calculated according to Jaccard’s index. A dendrogram showing the genetic stability between the three generations’ individuals and the mother plant was constructed using UPGMA (unweighted pair group method with arithmetic average) through CAP 1.2 software [57].

## 3. Results and Discussion

### 3.1. In Vitro Propagation

**Figure 1:**
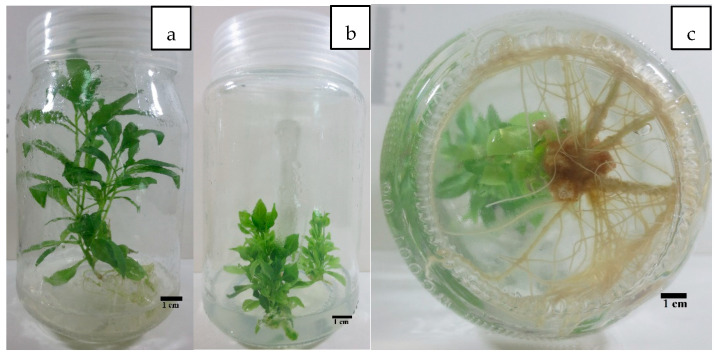
In vitro plant micropropagation protocol of *L. schweinfurthii*: (**a**) aseptic seedling; (**b**) shoot formation after five weeks of culture on MS medium supplemented with 0.4 mg L^−1^ Kinetin; (**c**) roots formed on MS medium fortified with 0.4 mg L^−1^ IBA (indole-3-butyric acid).

During the study of in vitro seed germination of *L. schweinfurthii*, the percentage of microbial contamination based on the method of sterilization used in culture media was 16.67%, and the maximum percentage of germination in non-contaminated cultures was 30% (Figure 1). Although due to the plant’s relatively low germination percentage, it was noticed that the germinated plants had plenty of leaves, convergent nodes, and elongated stems. The low growth rate reflected the low spread of the plant across large areas in the wildlife, which means that the in vitro multiplication of the plant is of great significance.

In an attempt for intensive plant micropropagation, nodal segments of sterilized germinated seedlings were cut and transferred into full-strength MS medium fortified with different concentrations of BA, Kin, and NAA as explained in Table 2. Strong variability was obtained in the number of leaves and shoot length of regenerated plantlets after 5 weeks of culture.

The highest significant results of shootlet length were observed in plantlets produced in MS medium with 0.4 mg L^−1^ kinetin, while 0.4 BA, 0.4 kinetin, and 0.2 BA + 0.2 Kin (in mg L^−1^) were recorded as highly significant in the number of distinct nodes (Figure 1). Although 0.4 mg L^−1^ kinetin was non-significant in other variables, with most treatments used it was the best in terms of the average number of leaves at approximately 26 leaves per regenerated plant. Moreover, it was second (1.86 plantlets/nodal segment) after 0.2 + 0.2 mg L^−1^ Kin + BA in terms of the number of plantlets regenerated per inoculated cut (2 plantlets/ nodal segment).

In vitro propagation of plants depends mainly on the addition of cytokinins to culture media and, sometimes, in addition to a lower concentration of auxins [58]. Two cytokinins (BA and Kin) and one auxin (NAA) were used for multiple shoot formations from nodal segments of *L. schweinfurthii*. The lower concentrations of cytokinins (BA or Kin) were the best in all determined variables, such as the number of plantlets, nodes, leaves, and shoot length. In the present study, a reduction in shoot proliferation by increasing benzyl-adenine or kinetin in the culture medium was noticed. Furthermore, similar results were observed when combinations between both growth regulators were added but with a total concentration the same as the concentration of only one of them. This allows saying that shoot formation in *L. schweinfurthii* may depend more on the concentration of the hormone than its type. These results are different from results obtained in the micropropagation of *Magnolia sirindhorniae, Eryngium alpinum,* and *Argania spinosa*. Shoots of *M. sirindhorniae* were optimally induced in a half-strength MS medium supplemented with a combination of BA, NAA, and gibberellic acid (GA_3_) with higher concentrations, i.e. 2.0 + 0.1 + 2.0 mgL^−1^, respectively [7]. A solid MS medium combined with BA, IAA, and GA_3_ was successful in shoot proliferation of *E. alpinum* [8]. Moreover, the highest adventitious shoots of the endangered plant, *A. spinosa,* were observed on MS medium containing 1 mg L^−1^ BA and 2 mg L^−1^ GA_3_ [59].

**Figure 2:**
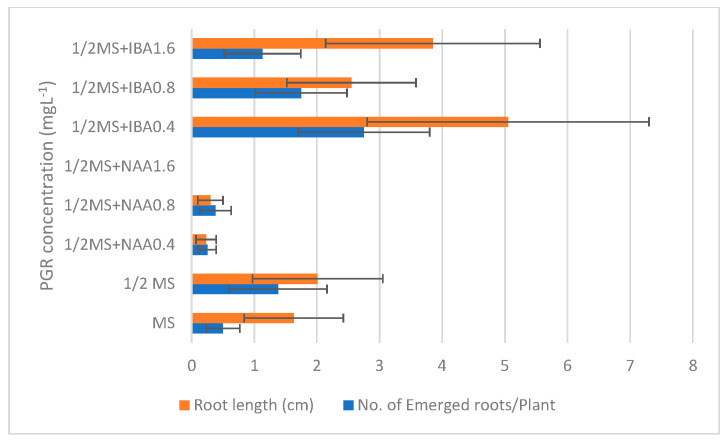
In vitro rooting of *L. schweinfurthii* shoots on MS medium fortified with different auxins. Pairwise comparison showed no significant differences between treatments at *p* ≤ 0.05 using the Holm–Sidak method. Eight replicates were used in each treatment. PGR, plant growth regulators; MS, full-strength MS salts; 1/2MS, half MS salts; IBA, indole-3-butyric acid; NAA, naphthalene acetic acid.

For completing in vitro micropropagation of the studied species, shootlets of the plant were transferred firstly to full- and half-strength MS media without growth regulators for root initiation. It was noticed that the number and length of roots that emerged in 1/2 MS were better than in the full-strength MS. Therefore, the experiment was repeated with the same treatments in addition to adding NAA and IBA to half-strength MS for rooting enhancement. It was found that increasing NAA concentration to medium reversely affected rooting production. Otherwise, the addition of 0.4 mg L^−1^ IBA enhanced the number of roots and the root length but with non-considerable significance with other treatments according to pairwise comparison using the Holm–Sidak method (Figure 1). Although the highest mean of the number of roots emerged per plant and long roots obtained in IBA treatments, not all eight shootlets showed a rooting response to the treatment. This led to a high standard error in several treatments and hid the significant differences between the different IBA concentrations used (Figure 2). However, the IBA treatments showed significantly better root formation and enhancement than NAA treatments.

In this study, half-strength MS medium with NAA and IBA were used for root stimulation, and IBA was the best for root formation enhancement. The results were consistent with other studies where IBA stimulated sufficient root induction in several species including *Cardiospermum halicacabum* [60], *Dorem ammoniacum* [61], *Achyranthes aspera* [62], and *Prunus armeniaca* L. [63].

### 3.2. Genetic Stability of Micropropagated Plantlets

For determining the genetic stability in the suggested micropropagation protocol, RAPD, ISSR, and SDS-PAGE analyses were performed to compare between the in vitro mother plant and its micropropagated plantlets, which resulted from using MS medium fortified with 0.4 mg L^−1^ BA for three generations and two individuals from each generation. Among the 20 primers screened (10 RAPD and 10 ISSR), only 12 primers produced clear and detectable amplified DNA fragments and were used in further PCR analysis. 

**Figure 3:**
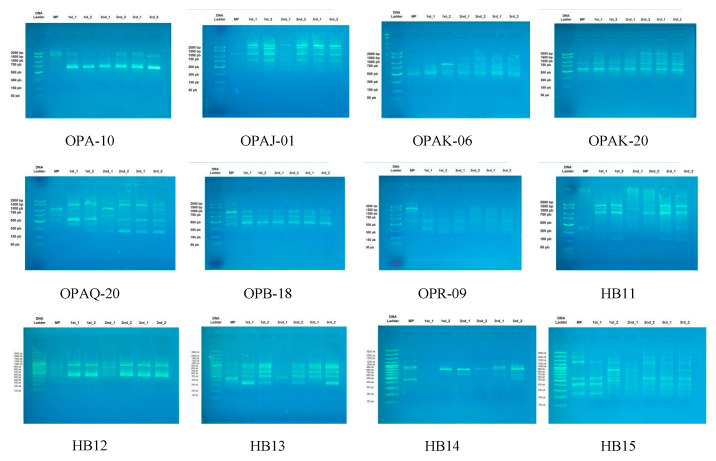
RAPD and ISSR profiles with primers mentioned in Table 1 of three micropropagated generations of *L. schweinfurthii* compared to the mother plant. MP, mother plant; 1st, first-generation plantlets; 2nd, second-generation plantlets; 3rd, third-generation plantlets.

With seven RAPD primers, 29 DNA fragments (a total of 137 scorable bands) were amplified in the mother plant and its three generations plantlets. Jaccard’s similarity coefficient, ranging between 0.36 and 0.56, was obtained. The second and third generations showed a similarity of 0.48 and 0.52 to the mother plant, respectively. The highest polymorphism of 100% was observed in fragments amplified with OPA10 and OPAJ01 primers, while the lowest of 50% was in the amplified fragments using OPB18 primer. Only eight monomorphic fragments out of 29 DNA fragments were recorded. Furthermore, 28 DNA fragments (a total of 107 scorable bands) were amplified using five ISSR primers, while a similarity of 0.33-0.70 was recorded. The highest similarities to the mother plant were in the first (0.66) and third (0.55) generations (Figure 3). A higher polymorphism was observed over RAPD, where the lowest was 75% in the HB13 and HB14 primers and the highest was 100% in the HB11 primer. Out of 28 DNA fragments amplified with five ISSR primers, only four fragments were monomorphic.

**Figure 4:**
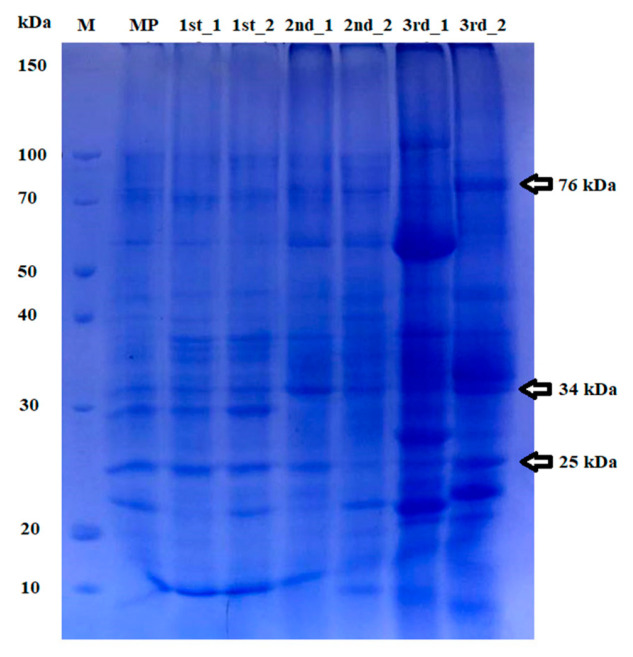
SDS-PAGE analysis of total protein bans extracted from three micropropagated generations of *L. schweinfurthii* compared to the mother plant. M, marker; MP, mother plant; 1st, first-generation plantlets; 2nd, second-generation plantlets; 3rd, third-generation plantlets showing three of the monomorphic polypeptides detected.

In SDS-PAGE analysis, sixteen polypeptides were separated with a similarity between 0.54 and 0.82. The first and second generations showed high similarity to the mother plant of 0.68 and 0.74, respectively (Figure 4). Half of the separated polypeptides were monomorphic, as they were found in all protein extracts. It was noticed also that there were two unique polypeptides of 82 and 108 KDa that were separated only in a plant in the third generation (3rd_1). The expressed protein showed uniformity between the mother plant and most of the plant individuals studied. On the contrary, only eight polypeptides were separated from *L. schweinfurthii* seed proteins in the study by El-Ghamry et al. [64].

**Figure 5:**
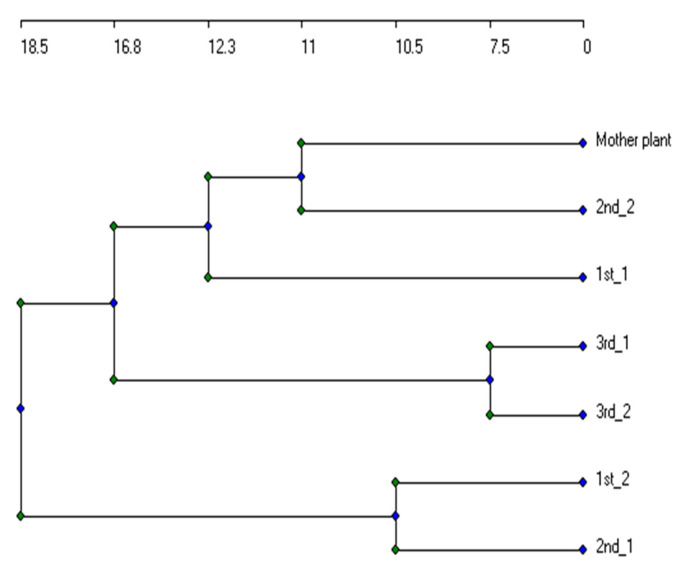
UPGMA dendrogram based on data generated from biochemical and molecular markers, showing the genetic linkage distance among the different micropropagated plantlets in different generations of *L. schweinfurthii*. MP, mother plant; 1st, first-generation plantlets; 2nd, second-generation plantlets; 3rd, third-generation plantlets.

Three matrices of RAPD, ISSR, and SDS-PAGE were merged and analyzed to show the clonal fidelity of the DNA and protein levels together. The dendrogram of genetic distances among the in vitro and micropropagated plants based on amplified DNA fragments generated by RAPD and ISSR primers and polypeptides separated in SDS-PAGE is shown in Figure 5. The distances in the dendrogram revealed that the first and third generations of the first plant individuals (1st_1 and 3rd_1) were more similar than the second generation (2nd_1). Furthermore, the second generation of the second individual (2nd_2) was more similar to the mother plant than the first (1st_2) and third (3rd_2) generations. The results showed that the generation that was more similar to the in vitro plants had the higher Jaccard’s similarity coefficient which ranged between 0 (completely different) and 1 (identical). The first micropropagated generation showed a higher similarity coefficient to the mother in vitro plants of 0.56–0.58. On the other hand, the second generation showed a similarity coefficient of 0.44–0.61, while the third one showed a similarity coefficient of 0.52–0.56 (Table 3). It was also obtained that the conditions of propagation in this study lowered the tendency of the plants to be genetically stable.

It is necessary after micropropagation to check the genetic uniformity of micropropagated plantlets [65]. Two PCR-based techniques (RAPD and ISSR) and a biochemical marker technique (SDS-PAGE) were used in the present study to test the genetic stability and polypeptide content because of their rapidity, simplicity, and effectiveness as well as the fact that they do not need prior information about the DNA sequence [66]. Moreover, the use of different markers in parallel provides better opportunities for genetic alteration identification between different clones [67]. The molecular markers were not affected by external environmental factors which, consequently, accurately detected the genetic variability among the plant clones [68]. The advantage of using both biochemical and molecular markers is the ability to give an account of the expression stability level of the DNA regarding the variability that occurred in the plant genome. In the present investigation, it was concluded that molecular and biochemical markers are equally important for genetic analysis and for the evaluation of the amount of genetic variability among the different micropropagated plantlets of *L. schweinfurthii*. In addition, Osman et al. [69] determined the genetic relationship between several species of *Zea mays* and *Sorghum* using SDS-PAGE of seed protein as well as RAPD-PCR markers.

In the present analysis, SDS-PAGE revealed the high stability of expressed proteins in the micropropagated plantlets compared to the amplified DNA fragments assessed by RAPD- and ISSR-PCR techniques. This indicates that it was supposed to have modifications in plantlet DNA, especially in the non-coding region. This effect may be related to the PGR used in micropropagation, as it was noticed that 6-benzyl adenine affects DNA and causes mutations [70]. In a study by Alizadeh and Singh [71], the similarity coefficient was 1 (in both RAPD and ISSR) in most clones, although there were low coefficients of 0.53 (RAPD) and 0.63 (ISSR) recorded in some clones of *Vitis* spp. micropropagated plantlets. This also raises the idea of the effects of PGRs and the cultivation conditions on the genetic stability of cloned plants.

### 3.3. Phenolic and Flavonoid Content Estimation

The phenolic and flavonoid contents of the micropropagated plant leaves’ extract were estimated spectrophotometrically in terms of gallic acid and catechin equivalence (GAE: gallic acid equivalent; CE: catechin equivalent) at 750 and 510 nm, respectively. Three replicates of different concentrations of gallic acid and catechin (10, 20, 30, 40, 50, 100, 150, 200, and 300 µg ml^−1^) were used to deduce the standard curves for determination of phenolic and flavonoid content, respectively. The generated equation for the gallic acid standard curve was $y=0.0043x+0.0019\;\left(R^{2}=0.9995\right)$. Furthermore, the generated one for the catechin standard curve was $y=0.0034x-0.0039\;\left(R^{2}=0.9993\right)$. The result obtained from the total phenolic content estimation of the in vitro leaves’ extracts was 11.53 mg GAE g^−1^ DW. However, the total flavonoid content was estimated as 12.99 mg CE g^−1^ DW.

From the rich plant sources of phenolics, *Acacia nilotica*, *Acacia catechu,* and *Albizia lebbeck* contain 80.63, 78.12, and 66.23 mg GAE, respectively [72]. Moreover, higher phenolic contents were estimated in the fruits of *Solanum indicum* and *S. surattense* of 250.4–289.5 mg GAE g^−1^ DW [73]. Despite the relatively lower total phenolics detected in this study, the global problem of food shortage necessitates the search for nutritional alternatives as well as nutritional supplements that preserve human health and vitality. On the other hand, the production of the active substance in vitro will remain the most appropriate solution that saves time and effort, especially due to the decline of global cultivated land and climate risks.

### 3.4. HPTLC Analysis

**Figure 6:**
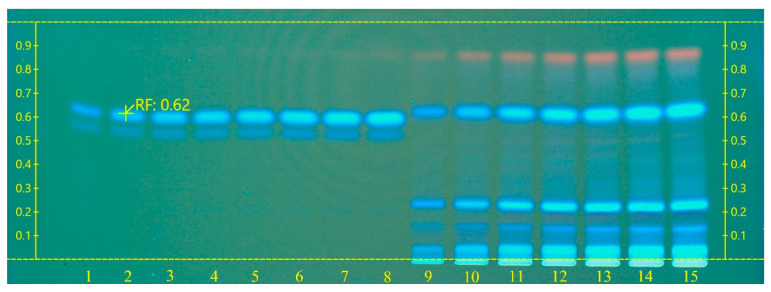
HPTLC chromatogram of *L. schweinfurthii* micropropagated leaves’ extract against ferulic acid standard captured at 366 nm. Tracks 1–8: ferulic acid, 400 µg ml^−1^ of volume 2–9 µL; Tracks 9–15: dry leaves’ extract of volume 2, 4, 6, 8, 10, 12, and 14 µL.

During the estimation of ferulic acid in dry leaves’ extract, the retardation factor (Rf) of the 400 µg ml^−1^ standard was 0.62 (Figure 6). The eight reference volumes (2–9 µL) of the standard were used to generate a linear calibration curve. The linear equation obtained was *y* = 5.601 × 10^−8^*x* *where R* = 95.21%, and the coefficient of variation (CV) was 11.77%. Only four of the seven different volumes of dry leaves’ extract samples (2, 4, 6, and 8 µL) were detected in the calibration range (Figure S5). The final results showed that the mean of ferulic acid content in the three samples within the calibration range was 45.52 mg g^−1^ DW where the CV = 1.19% (Table 4). The HPTLC method was simple, reproducible, and sensitive in the separation and determination of ferulic acid. It was used to estimate ferulic acid in *Lycopodium clavatum* [74], *Setaria italica* [75], and *Ricinus communis* Linn. [76].

### 3.5. Antioxidant Activities

The results obtained from the antioxidant assay revealed that 0.43 mg mL^−1^ of the in vitro leaves’ extract were required to scavenge half of the DPPH stable radicals (IC_50_). However, 1.99 mg mL^−1^ of the leaves’ extract were required to scavenge half of the stable ABTS free radicals (Table 4). According to plotting the inhibitory effect, the sensitivity and efficiency of the DPPH assay were higher than the ABTS assay. On the other hand, only 107.57 and 94.71 µg ml^−1^ of black pepper extracts were required to scavenge half of the DPPH and ABTS stable radicals, respectively [55].

## 4. Conclusions

In this study, we successfully established a suitable, rapid, and efficient protocol for in vitro micropropagation of *L. schweinfurthii* from nodal segments. Reproducible genetic and biochemical techniques were performed to determine the stability of plant genome and expressed proteins in regenerated in vitro plants. The importance of the leaves’ extract was proven through the content and activity. This protocol should be useful in future studies for in vitro secondary metabolite production from this plant.

## Figures and Tables

**Table 1:** Sequences and annealing temperatures of primers used for the RAPD and ISSR analysis of *L. schweinfurthii*.

Primer Name		Sequence	Annealing Temperature (°C)
RAPD primers	OPA-10	5′-GTGATCGCAG-3′	40.5
	OPAJ-01	5′-ACGGGTCAGA-3′	43
	OPAK-06	5′-TCACGTCCCT-3′	42
	OPAK-20	5′-TGATGGCGTC-3′	41
	OPAQ-20	5′-GTGAACGCTC-3′	40.5
	OPB-18	5′-CCACAGCAGT-3′	42
	OPR-09	5′-TGAGCACGAG-3′	42
ISSR primers	HB11	5′-GTGTGTGTGTGTCC-3′	54
	HB12	5′-CACCACCACGC-3′	50.9
	HB13	5′- GAGGAGGAGGC-3′	48
	HB14	5′-CTCCTCCTCGC-3′	48
	HB15	5′-GTGGTGGTGGC-3′	50.9

**Table 2:** Effect of different concentrations of BA, Kin, and NAA on micropropagation of *L. schweinfurthii* from nodal cuttings.

Treatment (mg L^−1^)			Number of Plantlets	Number of Distinct Nodes	Number of Leaves	Shootlet Length (cm)
BA	Kin	NAA				
-	-	-	1.00 ± 0.00 ^abc^	1.00 ± 0.00 ^abc^	4.14 ± 0.46 ^c^	0.56 ± 0.03 ^b^
0.4	-	-	1.43 ± 0.48 ^abc^	3.29 ± 0.89 ^ab^	20.71 ± 3.66 ^ad^	1.24 ± 0.40 ^b^
0.8	-	-	1.00 ± 0.22 ^abc^	1.71 ± 0.47 ^abc^	5.86 ± 2.19 ^cd^	0.83 ± 0.17 ^b^
1.6	-	-	1.14 ± 0.14 ^abc^	1.29 ± 0.18 ^abc^	7.14 ± 2.44 ^bcd^	0.71 ± 0.19 ^b^
3.2	-	-	0.29 ± 0.18 ^c^	0.43 ± 0.30 ^b^	1.86 ± 1.70 ^c^	0.20 ± 0.13 ^b^
-	0.4	-	1.86 ± 0.46 ^ab^	5.86 ± 0.91 ^a^	26.00 ± 4.34 ^a^	2.83 ± 0.39 ^a^
-	0.8	-	0.86 ± 0.14 ^abc^	1.71 ± 0.52 ^abc^	8.00 ± 2.17 ^bcd^	0.93 ± 0.25 ^b^
-	1.6	-	0.14 ± 0.14 ^c^	0.14 ± 0.14 ^b^	0.43 ± 0.43 ^c^	0.07 ± 0.07 ^b^
-	3.2	-	0.86 ± 0.14 ^abc^	1.00 ± 0.22 ^abc^	8.14 ± 1.97 ^bcd^	0.64 ± 0.13 ^b^
0.2	0.2	-	2.00 ± 0.44 ^a^	3.86 ± 1.12 ^a^	22.14 ± 4.49 ^ab^	1.23 ± 0.20 ^b^
0.4	0.4	-	1.29 ± 0.18 ^abc^	2.14 ± 0.55 ^abc^	12.71 ± 4.20 ^a^	1.11 ± 0.24 ^b^
0.8	0.8	-	1.43 ± 0.30 ^abc^	2.14 ± 0.46 ^abc^	12.43 ± 3.61 ^a^	1.14 ± 0.31 ^b^
1.6	1.6	-	1.00 ± 0.22 ^abc^	1.71 ± 0.61 ^abc^	8.29 ± 2.73 ^bcd^	0.91 ± 0.26 ^b^
0.2	0.2	0.2	0.57 ± 0.37 ^c^	1.00 ± 0.66 ^abc^	6.00 ± 3.93 ^cd^	0.51 ± 0.34 ^b^
0.4	0.4	0.4	0.43 ± 0.20 ^c^	1.29 ± 0.97 ^abc^	4.43 ± 2.41 ^c^	0.63 ± 0.38 ^b^

Pairwise comparison was conducted according to the Holm–Sidak method at *p* ≤ 0.05. Seven replicates were used for each treatment; BA, 6-benzyl adenine; Kin, kinetin; NAA, naphthalene acetic acid. The letters a, b, c, and d represent the pairwise comparison and the significance between treatments.

**Table 3:** Jaccard’s similarity coefficient concerning similarities in DNA fragments generated in RAPD and ISSR analyses and protein polypeptides through SDS-PAGE.

	Mother Plant	1st_1	1st_2	2nd_1	2nd_2	3rd_1	3rd_2
Mother Plant							
1st_1	0.5806						
1st_2	0.5593	0.5738					
2nd_1	0.4364	0.4821	0.5714				
2nd_2	0.614	0.6271	0.5	0.54			
3rd_1	0.5625	0.6508	0.5556	0.4912	0.6333		
3rd_2	0.5246	0.5645	0.5424	0.4717	0.569	0.7368	

**Table 4:** A summary of the results of the total phenolic content, total flavonoid content, ferulic acid content, and antioxidant activity of *micropropagated* L. schweinfurthii dried leaves.

Contents and Antioxidant Capacity	Obtained Results
Total phenolic content	11.53 GAE g^−1^ DW
Total flavonoid content	12.99 CE g^−1^ DW
Ferulic acid content	45.52 mg g^−1^ DW
IC_50_ with DPPH analysis	0.43 mg mL^−1^
IC_50_ with ABTS^+^ analysis	1.99 mg mL^−1^

## Supplementary Materials

The following are available online at https://www.mdpi.com/article/10.3390/plants10102089/s1, Figure S1: Jazirat Al-Kawm Al-Akhdar (the green islet) which is located in Burullus Lake (northern of Nile Delta) in Egypt showing the populations of *Lycium schweinfurthii*. Figure S2: The blooming of *L. schweinfurthii* during the spring season. Figure S3: *L.schweinfurthii* branch showing leaves and immature fruits. Figure S4: Ripe fruits of *L. schweinfurthii*. Figure S5: Calibration range of the HPTLC analysis of micropropagated dry leaves’ extract samples against a reference of ferulic acid 400 µg ml^−1^.
