# Supplementary material for: Genetic Stability, Phenolic, Flavonoid, Ferulic Acid Contents, and Antioxidant Activity of Micropropagated Lycium schweinfurthii Plants
Source: Plants (Basel). 2021 Oct 1;10(10):2089. doi: 10.3390/plants10102089 (PMC8540154; doi:10.3390/plants10102089)
Supplement: Supplementary file 1 [file plants-10-02089-s001.zip › plants-1392765-supplementary.pdf]

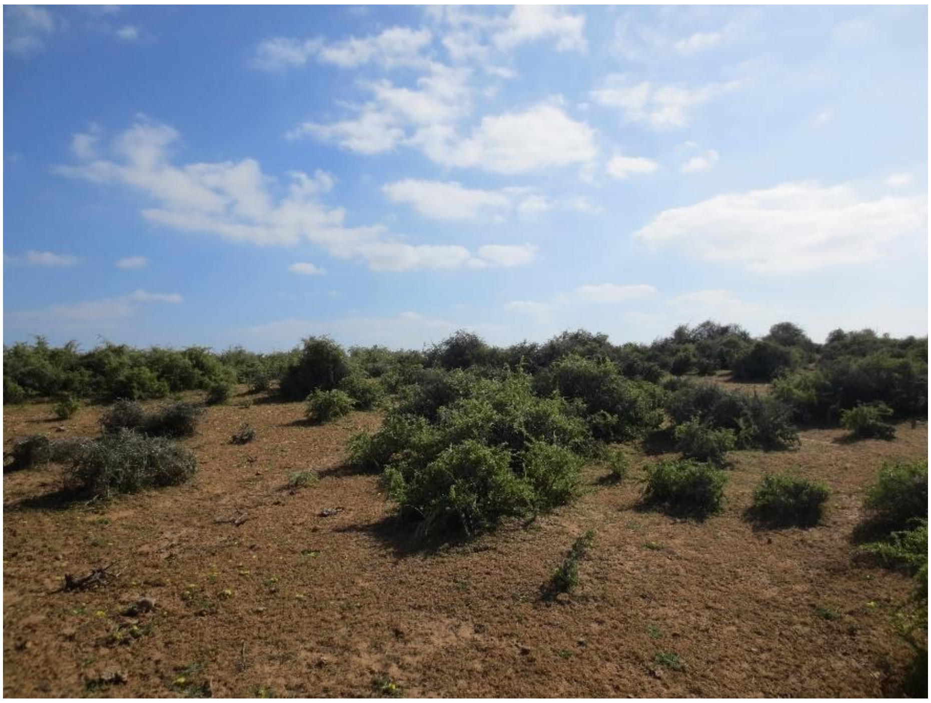

Figure S1. Jazirat Al-Kawm Al-Akhdar (the green islet) which is located in Burullus Lake (northern of Nile Delta) in Egypt showing the populations of *Lycium schweinfurthii.*

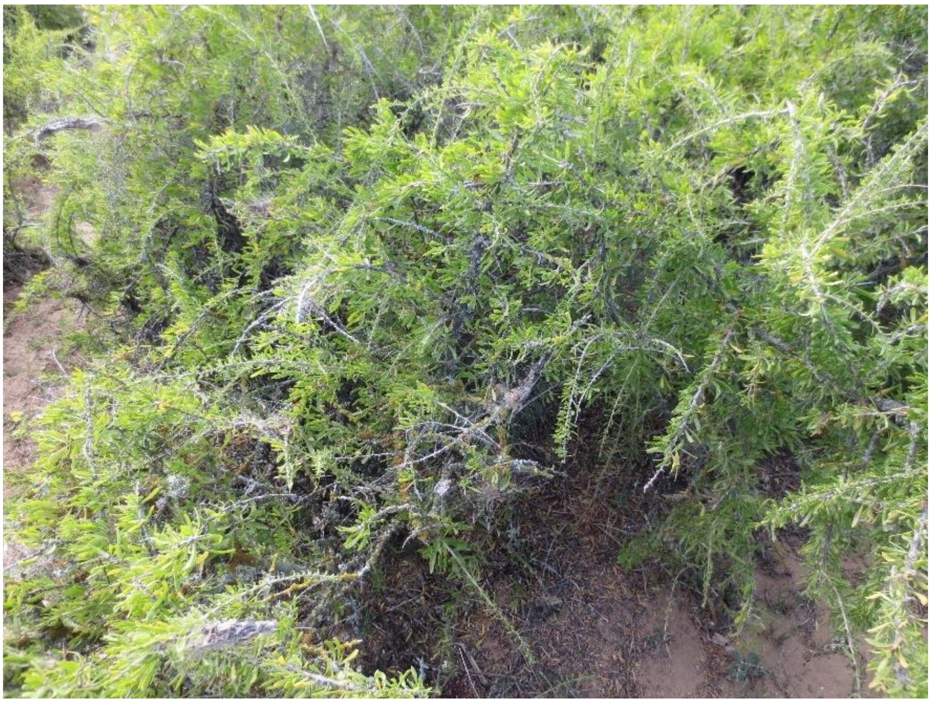

Figure S2. The blooming of *L. schweinfurthii* during the spring season.

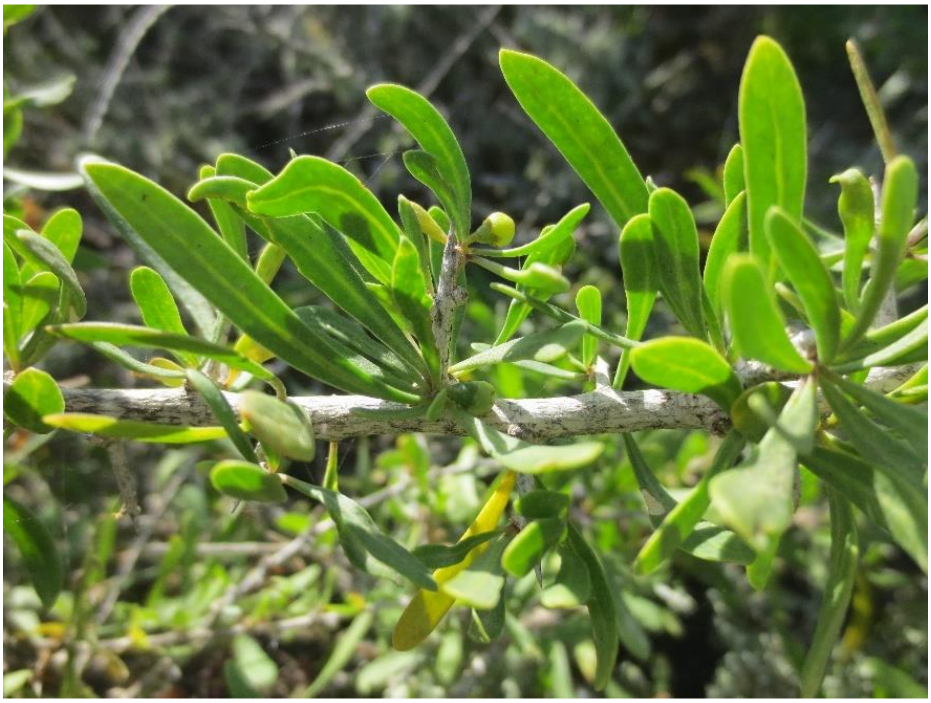

Figure S3. *L.schweinfurthii* branch showing leaves and immature fruits.

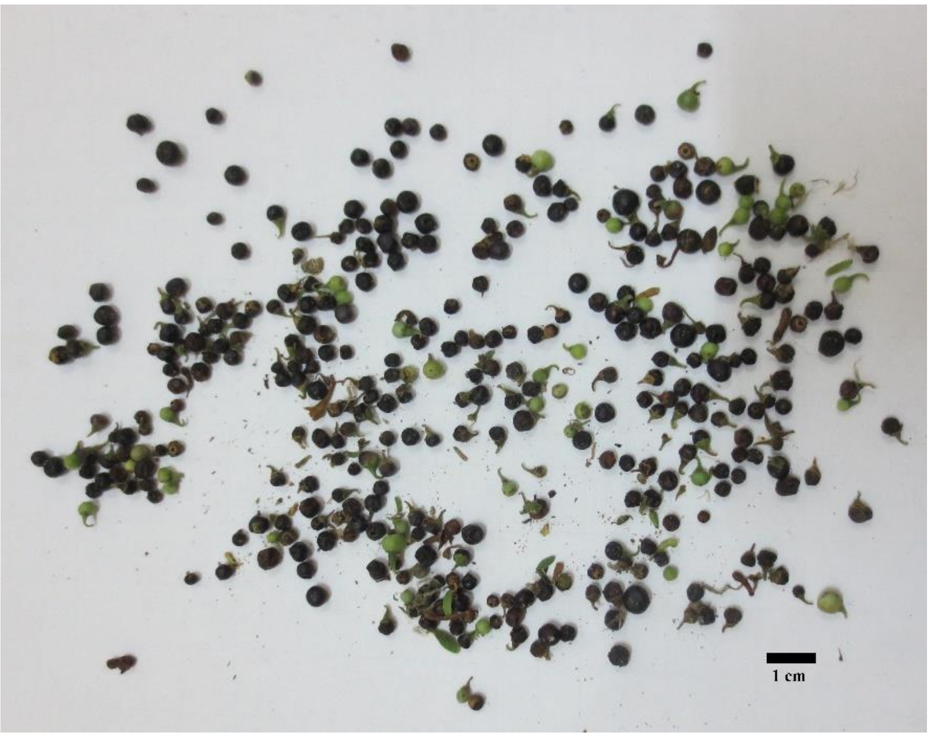

Figure S4. Ripe fruits of *L. schweinfurthii*.

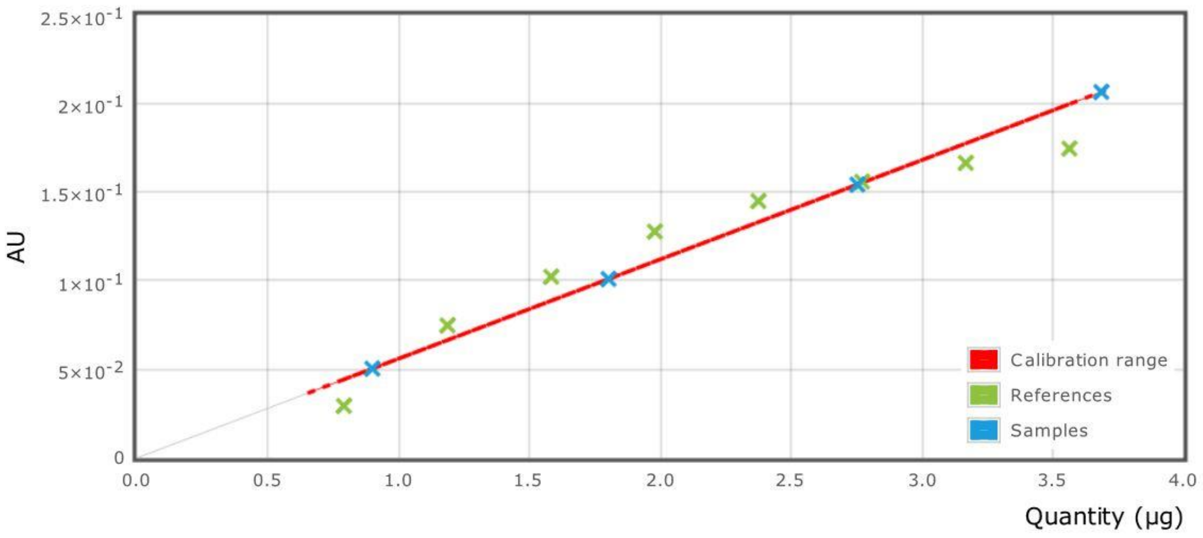

Figure S5. Calibration range of the HPTLC analysis of micropropagated dry leaves' extract samples against a reference of ferulic acid 400 µg ml^{-1}.
